# Amelioration of Fibrosis via S1P Inhibition Is Regulated by Inactivation of TGF-β and SPL Pathways in the Human Cornea

**DOI:** 10.3390/ijms25126560

**Published:** 2024-06-14

**Authors:** Sarah E. Nicholas, Sandip K. Basu, Nawajes Mandal, Dimitrios Karamichos

**Affiliations:** 1 North Texas Eye Research Institute, University of North Texas Health Science Center, Fort Worth, TX 76107, USA; sarah.nicholas@unthsc.edu; 2 Department of Pharmaceutical Sciences, University of North Texas Health Science Center, Fort Worth, TX 76107, USA; 3 Department of Ophthalmology, University of Tennessee Health Science Center, Memphis, TN 38163, USA; sbasu8@uthsc.edu (S.K.B.); nmandal@uthsc.edu (N.M.); 4 Department of Anatomy and Neurobiology, University of Tennessee Health Science Center, Memphis, TN 38163, USA; 5 Department of Pharmacology and Neuroscience, University of North Texas Health Science Center, Fort Worth, TX 76107, USA

**Keywords:** corneal fibrosis, fibrotic rescue, fibrotic prevention, TGF-β, S1P, sphingosine kinase inhibitor 2, SphK I_2_, LTBP, SMAD

## Abstract

Human corneal fibrosis can lead to opacity and ultimately partial or complete vision loss. Currently, corneal transplantation is the only treatment for severe corneal fibrosis and comes with the risk of rejection and donor shortages. Sphingolipids (SPLs) are known to modulate fibrosis in various tissues and organs, including the cornea. We previously reported that SPLs are tightly related to both, transforming growth factor beta (TGF-β) signaling and corneal fibrogenesis. The aim of this study was to investigate the effects of sphingosine-1-phosphate (S1P) and S1P inhibition on specific TGF-β and SPL family members in corneal fibrosis. Healthy human corneal fibroblasts (HCFs) were isolated and cultured in EMEM + FBS + VitC (construct medium) on 3D transwells for 4 weeks. The following treatments were prepared in a construct medium: 0.1 ng/mL TGF-β1 (β1), 1 μM sphingosine-1-phosphate (S1P), and 5 μM Sphingosine kinase inhibitor 2 (I_2_). Five groups were tested: (1) control (no treatment); rescue groups; (2) β1/S1P; (3) β1/I_2_; prevention groups; (4) S1P/β1; and (5) I_2_/β1. Each treatment was administered for 2 weeks with one treatment and switched to another for 2 weeks. Using Western blot analysis, the 3D constructs were examined for the expression of fibrotic markers, SPL, and TGF-β signaling pathway members. Scratch assays from 2D cultures were also utilized to evaluate cell migration We observed reduced fibrotic expression and inactivation of latent TGF-β binding proteins (LTBPs), TGF-β receptors, Suppressor of Mothers Against Decapentaplegic homologs (SMADs), and SPL signaling following treatment with I_2_ prevention and rescue compared to S1P prevention and rescue, respectively. Furthermore, we observed increased cell migration following stimulation with I_2_ prevention and rescue groups, with decreased cell migration following stimulation with S1P prevention and rescue groups after 12 h and 18 h post-scratch. We have demonstrated that I_2_ treatment reduced fibrosis and modulated the inactivation of LTBPs, TGF-β receptors, SPLs, and the canonical downstream SMAD pathway. Further investigations are warranted in order to fully uncover the potential of utilizing SphK I_2_ as a novel therapy for corneal fibrosis.

## 1. Introduction

Corneal fibrosis is a leading cause of blindness worldwide, affecting over 10 million people [1]. Injury or trauma to the cornea can initiate resident keratocyte differentiation into myofibroblasts, causing high expression levels of α smooth muscle actin (αSMA) and Collagen III, which can cause irregular deposition of extracellular matrix (ECM) components [2,3]. This disruption of the homeostatic corneal environment can cause cornea scarring, which can result in vision loss. While the corneal clarity is maintained by orchestrated signaling cascades, the specific mechanisms driving corneal fibrosis are very complex and still not well understood [1,4,5,6]. Unfortunately, for severe corneal scars, corneal transplantation is often the only viable option for those suffering from this condition.

Previous studies have investigated the role of transforming growth factor beta (TGF-β) isoforms in cells and fibrotic tissues [7,8,9,10,11,12,13]. The three isoforms found in humans share approximately 80% homology but their actions on cells and tissues vary greatly. Our group and others have found that the TGF-β1 isoform induces corneal fibrosis, whereas the TGF-β3 isoform is known for its anti-fibrotic abilities [4,14,15,16,17,18,19,20,21,22,23,24]. Various signaling cascades are initiated when TGF-β isoforms bind to TGF-β receptors [7,25,26,27]. The activation of latent TGF-β is regulated largely by latent TGF-β binding proteins (LTBPs). LTBPs 1–4 are secreted by various cells and tissues [28,29,30,31], including the cornea; however, their role in the cornea is not currently well understood.

Sphingosine 1-phosphate (S1P) is a pleiotropic bioactive lipid mediator that is formed from the catalysis of sphingosine kinases (SphK1 and SphK2). Sphingolipids (SPLs), such as S1P, bind to S1P receptors (S1PR1-5), inducing various cellular responses, and have been implicated in many studies as key regulators of fibrosis in various tissues and organs [32,33,34,35,36,37,38,39,40,41,42,43,44,45,46,47,48], including the cornea [26,33,49,50,51,52,53,54,55,56].

**Figure 1:**
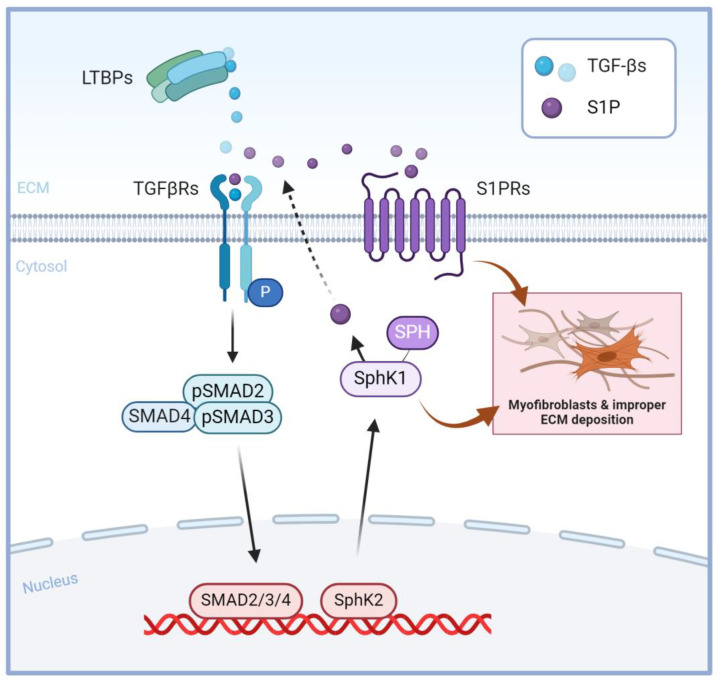
S1P and TGF-β signaling cross-talk: TGF-β receptor activation induces SMAD signaling cascades, resulting in the regulation of gene expression, including SphK regulation. Stimulation of SphK1 leads to the formation of S1P, which activates S1P receptors, inducing fibrotic cell responses. Created with Biorender.com.

In the cornea, the cross-talks between TGF-β and S1P signaling remain elusive; however, studies in other tissues suggest that TGF-β induces the activity of sphingosine kinase [45,57]. Several recent studies have demonstrated the fibrotic protective effects of TGF-β and S1P inhibition [58,59,60,61,62]. Figure 1 represents the “inside out” signaling of S1P and TGF-β, both autocrine and paracrine, as observed by our team and other researchers [34,35,63,64,65]. Our group recently investigated the interplay of S1P and SPHK I_2_ (I_2_; a selective inhibitor of SphK1) interactions with TGF-β signaling and downstream signaling in human corneal fibrosis [26]. We demonstrated differential regulation of TGF-βRII following S1P stimulation vs. I_2_ treatment. Additionally, S1P inhibition downregulated pSMAD2 and SMAD4 and showed similar signaling patterns as TGF-β3 treatment.

The present study sought to understand the role of SPLs and TGF-β signaling pathway members, their cross-talks, and downstream targets in corneal fibrosis. Future studies will reveal whether S1P inhibition can be tailored as a novel therapy for the management of corneal fibrosis.

## 2. Results

### 2.1. Latent Transforming Growth Factor Beta Binding Proteins (LTBPs)

**Figure 2:**
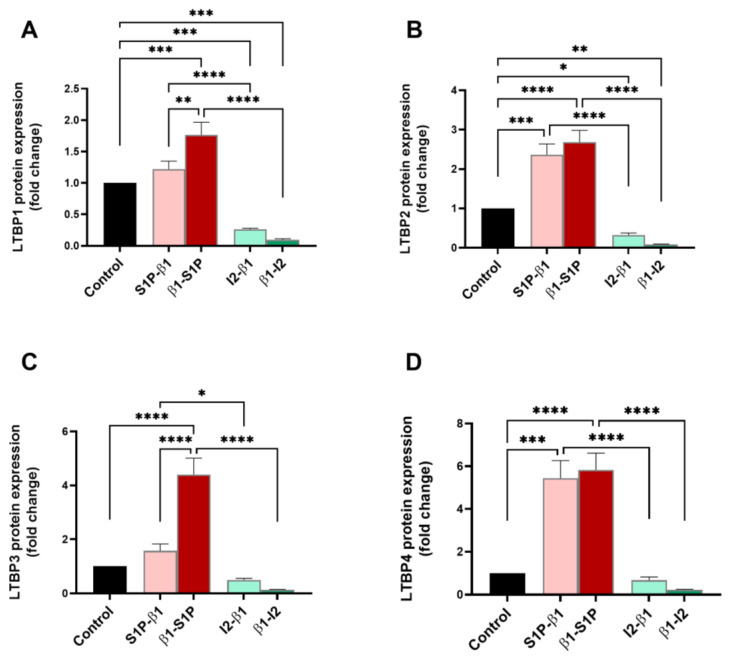
LTBPs 1–4 protein expressions in HCF 3D constructs in response to stimulation with S1P-β1 (S1P prevention), β1-S1P (S1P rescue), I_2_-β1 (I_2_ prevention), and β1-I_2_ (I_2_ rescue). (**A**) LTBP1 expression in HCFs (n = 3). (**B**) LTBP2 expression in HCFs (n = 3). (**C**) LTBP3 expression in HCFs (n = 3). (**D**) LTBP4 expression in HCFs (n = 3). One-way ANOVA; * *p* < 0.05, ** *p* < 0.01, *** *p* < 0.001, and **** *p* < 0.0001.

Latent TGF-β binding proteins-1 through -4 (LTBPs 1–4) are known activators of TGF-β and were investigated here for their protein expressions following treatment with all groups tested. LTBP1 expression was significantly upregulated by β1-S1P stimulation compared to the controls (*p* = 0.0005), S1P-β1 (*p* = 0.0048), and β1-I_2_ (*p* < 0.0001) treatments. In Figure 2A, I_2_-β1 stimulation led to a significant downregulation of LTBP1 compared to S1P-β1 treatment (*p* < 0.0001) and the controls (*p* = 0.006). LTBP2 expression was significantly upregulated by β1-S1P stimulation compared to the controls (*p* < 0.0001) and β1-I_2_ treatment (*p* < 0.0001) (Figure 2B). Figure 2B showed that treatment with I_2_-β1 caused a significant downregulation of LTBP2 compared to S1P-β1 treatment (*p* < 0.0001) and the controls (*p* = 0.0258). The expression of LTBP3 was significantly upregulated by β1-S1P stimulation compared to the controls (*p* < 0.0001), S1P-β1 (*p* < 0.0001), and β1-I_2_ (*p* < 0.0001) treatments (Figure 2C). The I_2_-β1 stimulation caused a significant downregulation of LTBP3 compared to S1P-β1 treatment (*p* = 0.0298) (Figure 2C). LTBP4 was significantly upregulated by β1-S1P stimulation compared to the controls (*p* < 0.0001) and β1-I_2_ treatment (*p* < 0.0001) (Figure 2D). Treatment with I_2_-β1 led to a significant downregulation of LTBP4 compared to S1P-β1 (*p* < 0.0001) treatment and the controls (Figure 2D).

### 2.2. Transforming Growth Factor Beta Receptors (TGF-βRs)

**Figure 3:**
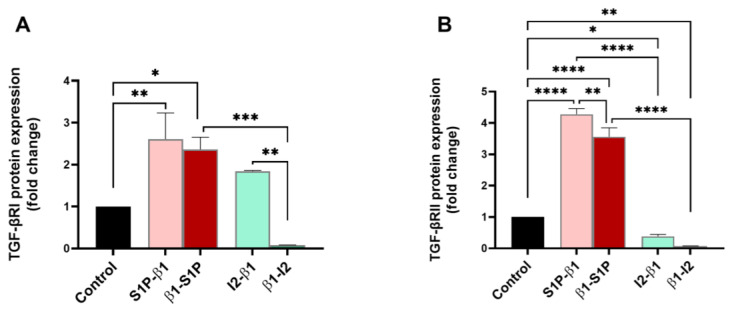
TGF-β receptors protein expressions in HCF 3D constructs in response to stimulation with S1P-β1 (S1P prevention), β1-S1P (S1P rescue), I_2_-β1 (I_2_ prevention), and β1-I_2_ (I_2_ rescue). (**A**) TGF-βRI expression in HCFs (n = 3). (**B**) TGF-βRII expression in HCFs (n = 3). One-way ANOVA; * *p* < 0.05, ** *p* < 0.01, *** *p* < 0.001, and **** *p* < 0.0001.

TGF-β receptors I and II (TGF-βRI and TGF-βRII), which are activated via binding with active TGF-β, were investigated for their protein expressions for all groups tested. TGF-βRI expression was significantly upregulated by stimulation with β1-S1P compared to the controls (*p* = 0.0106), β1-I_2_ (*p* = 0.0004), and S1P-β1 compared to the controls only (*p* = 0.0041) (Figure 3A). β1-I_2_ treatment caused a significant downregulation of TGF-βRI compared to I_2_-β1 (*p* = 0.0023; Figure 3A). TGF-βRII was significantly upregulated by the S1P-β1 treatment compared to the controls (*p* < 0.0001), β1-S1P (*p* = 0.0076), and I_2_-β1 (*p* < 0.0001) treatments (Figure 3B). β1-S1P stimulation significantly upregulated TGF-βRII compared to the controls (*p* < 0.0001) and β1-I_2_ treatment (*p* < 0.0001) (Figure 3B).

### 2.3. Canonical Downstream SMAD Pathway

**Figure 4:**
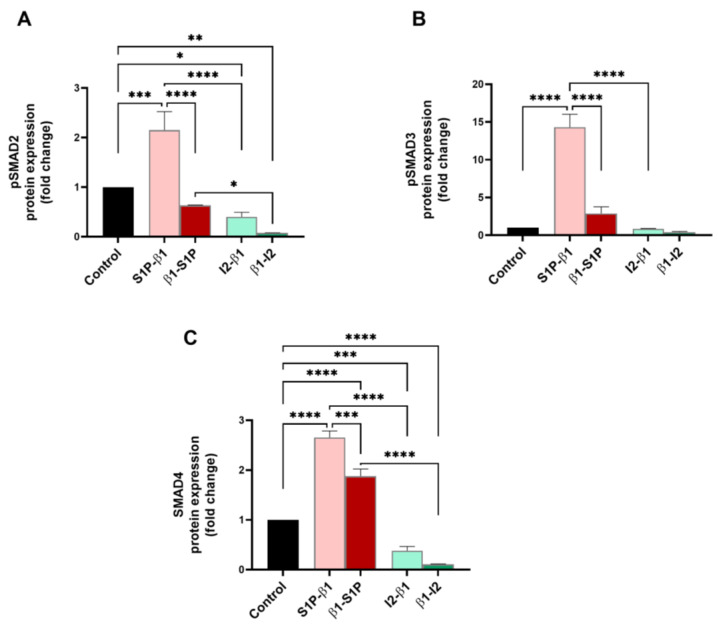
SMAD pathway protein expressions in HCF 3D constructs in response to stimulation with S1P-β1 (S1P prevention), β1-S1P (S1P rescue), I_2_-β1 (I_2_ prevention), and β1-I_2_ (I_2_ rescue). (**A**) pSMAD2 expression in HCFs (n = 3). (**B**) pSMAD3 expression in HCFs (n = 3). (**C**) SMAD4 expression in HCFs (n = 3). One-way ANOVA; * *p* < 0.05, ** *p* < 0.01, *** *p* < 0.001, and **** *p* < 0.0001.

SMADs 2–4, which are the main signal transducers for TGF-β receptors, were investigated for their protein expressions for all groups tested. Figure 4A shows that pSMAD2 expression was significantly upregulated by S1P-β1 stimulation compared to the controls (*p* = 0.0008), β1-S1P (*p* < 0.0001), and I_2_-β1 (*p* < 0.0001) treatments. Stimulation with β1-I_2_ led to a significant downregulation of pSMAD2 compared to the controls (*p* = 0.0035) and β1-S1P treatment (*p* = 0.0446) (Figure 4A). pSMAD3 expression was significantly upregulated by S1P-β1 compared to the controls (*p* < 0.0001), β1-S1P (*p* < 0.0001), and I_2_-β1 (*p* < 0.0001) treatments (Figure 4B). SMAD4 expression was significantly upregulated by S1P-β1 treatment compared to the controls (*p* < 0.0001), β1-S1P (*p* = 0.0002), and I_2_-β1 (*p* < 0.0001) treatments (Figure 4C). Stimulation with β1-S1P significantly upregulated SMAD4 compared to the controls (*p* < 0.0001) and β1-I_2_ treatment (*p* < 0.0001) (Figure 4C).

### 2.4. Sphingolipid Members Pathway

**Figure 5:**
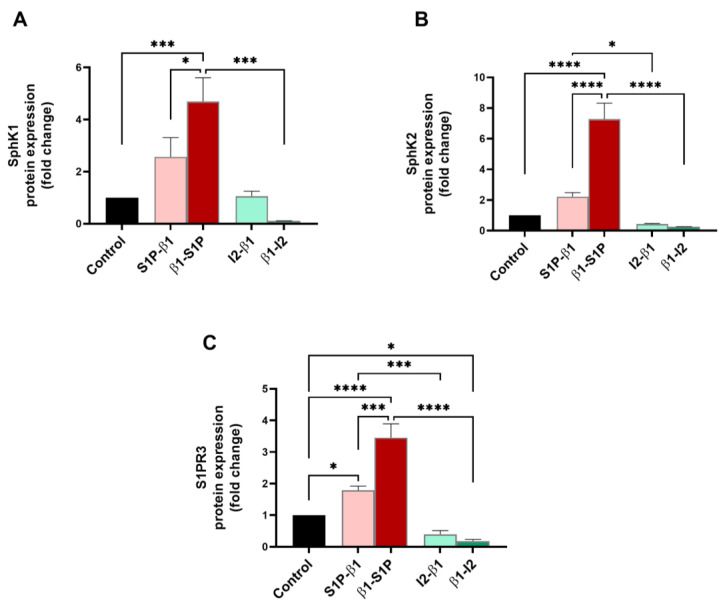
Sphingolipid pathway protein expressions in HCF 3D constructs in response to stimulation with S1P-β1 (S1P prevention), β1-S1P (S1P rescue), I_2_-β1 (I_2_ prevention), and β1-I_2_ (I_2_ rescue). (**A**) SphK1 expression in HCFs (n = 3). (**B**) SphK2 expression in HCFs (n = 3). (**C**) S1PR3 expression in HCFs (n = 3). One-way ANOVA; * *p* < 0.05, *** *p* < 0.001, and **** *p* < 0.0001.

Sphingolipid members (SphK1, SphK2, and S1PR3), which are known to transactivate the TGF-β pathway, were examined for their protein expressions for all groups tested. SphK1 expression was significantly upregulated by β1-S1P treatment compared to the controls (*p* = 0.0006), S1P-β1 (*p* = 0.0181), and β1-I_2_ stimulation (*p* = 0.001) (Figure 5A). Tge expression of SphK2 was significantly upregulated by β1-S1P compared to the controls (*p* < 0.0001), S1P-β1 (*p* < 0.0001), and β1-I_2_ (*p* < 0.0001) treatments (Figure 5B). The I_2_-β1 treatment led to a significant downregulation of SphK2 compared to S1P-β1 stimulation (*p* = 0.0259; Figure 5B). S1PR3 was significantly upregulated by the β1-S1P treatment compared to the controls (*p* < 0.0001), S1P-β1 (*p* = 0.0002), and β1-I_2_ (*p* < 0.0001) treatments (Figure 5C). Stimulation with I_2_-β1 caused a significant downregulation of S1PR3 compared to the S1P-β1 treatment (*p* = 0.0009; Figure 5C).

### 2.5. Fibrosis Markers

**Figure 6:**
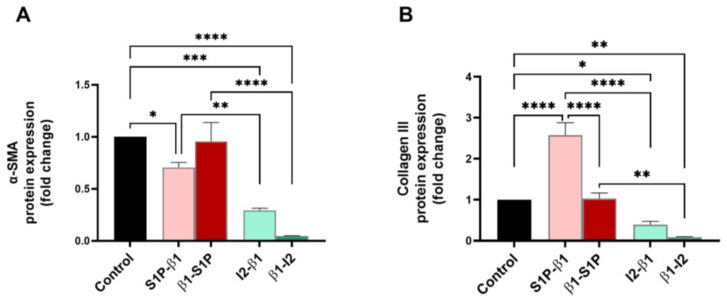
Fibrosis marker protein expressions in HCF 3D constructs in response to stimulation with S1P-β1 (S1P prevention), β1-S1P (S1P rescue), I_2_-β1 (I_2_ prevention), and β1-I_2_ (I_2_ rescue). (**A**) α-SMA expression in HCFs (n = 3). (**B**) Collagen III expression in HCFs (n = 3). One-way ANOVA; * *p* < 0.05, ** *p* < 0.01, *** *p* < 0.001, and **** *p* < 0.0001.

Corneal fibrosis markers, α-smooth muscle actin (α-SMA), and Collagen III were examined for their protein expressions for all groups tested. α-SMA expression was significantly downregulated by the I_2_-β1 treatment compared to the controls (*p* = 0.0001) and the S1P-β1 (*p* = 0.0062) treatment (Figure 6A). β1-I_2_ stimulation caused a significant downregulation of α-SMA compared to the controls (*p* < 0.0001) and β1-S1P (*p* < 0.0001) stimulation (Figure 6A). The expression of Collagen III was significantly downregulated with the β1-I_2_ treatment compared to the controls (*p* = 0.0018) and β1-S1P (*p* = 0.0014) stimulation (Figure 6B). S1P-β1 caused a significant upregulation of Collagen III compared to the control (*p* < 0.0001), β1-S1P (*p* < 0.0001), and I_2_-β1 (*p* < 0.0001) treatments (Figure 6B).

### 2.6. Effects of S1P and TGF-β Treatment Groups on HCF Cellular Migration

**Figure 7:**
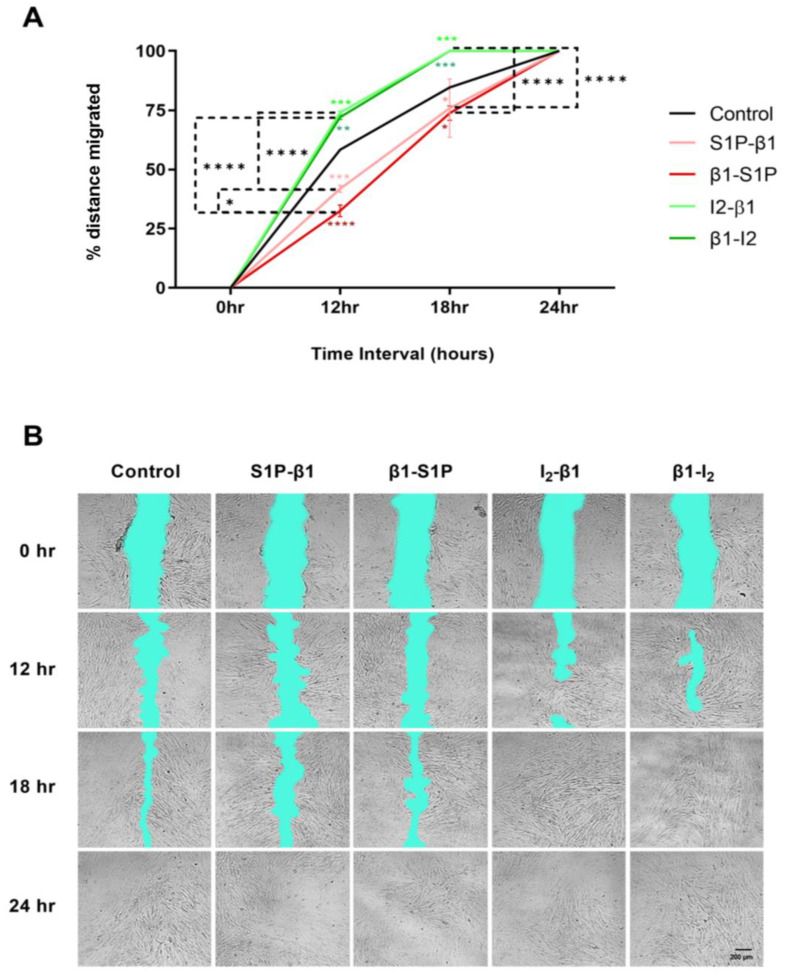
Effects of HCF cellular migration in response to stimulation with S1P-β1 (S1P prevention), β1-S1P (S1P rescue), I_2_-β1 (I_2_ prevention), and β1-I_2_ (I_2_ rescue) via the scratch assay. (**A**) Cell migration % quantification (n = 5). (**B**) Representative scratch assay images at 0, 12, 18, and 24 h post-scratch. Two-way ANOVA; * *p* < 0.05, ** *p* < 0.01, *** *p* < 0.001, and **** *p* < 0.0001.

Cellular migration of HCFs was examined in response to stimulation with all groups tested over a period of 24 h. Following 12 h of stimulation with the I2-β1 group, we observed significantly increased wound closure compared with the controls (*p* = 0.0006) and S1P-β1 (*p* < 0.0001; Figure 7A). Similarly, the β1-I_2_ group caused significantly increased wound closure after 12 h compared to the controls (*p* = 0.0024) and β1-S1P (*p* < 0.0001) stimulation (Figure 7A). After 18 h, we observed significantly increased wound closure with I_2_-β1 compared to the controls (*p* = 0.0008) and β1-S1P (*p* < 0.0001) and in β1_–_I_2_ compared to the controls (*p* = 0.0008) and β1-S1P (*p* < 0.0001) stimulation (Figure 7A). After 24 h, all treatment groups reached 100% wound closure (Figure 7A). Representative cell migration images with all groups tested over the course of 24 h are shown in Figure 7B.

## 3. Discussion

The mechanisms involving TGF-β and S1P in the cornea have largely remained a mystery due to the complexity of their signaling effects the lack of studies. Our group previously reported on the signaling expressions of SPLs, TGF-β members, canonical downstream SMAD, non-canonical downstream, and fibrotic markers in HCF 3D constructs treated with S1P, I_2_, TGF-β1, and TGF-3 [26]. Our current study demonstrated the impact of I_2_-induced fibrotic prevention and rescue modulated by SPLs and TGF-β family member signaling in HCF 3D constructs.

Inactive TGF-β isoforms are secreted from cells and are activated in covalent association with LTBP molecules [30,66]. Acosta et al., 2023, reported the presence of increased LTBP1 expression in murine corneal fibroblasts [16]. Another recent study found an increased expression of LTBP1 in the keratotomy wounds of mouse corneas [67]. LTBP2 dysregulation has been linked to eye diseases in numerous studies, including glaucoma [68,69,70,71,72,73,74,75,76,77]. Two studies reported LTBP1 and LTBP2 upregulation in the anterior segment of human tissues with pseudoexfoliation syndrome [78,79]. Similarly, De Maria et al., 2021, found LTBP2 and LTBP3 upregulation in the lens capsule and aqueous humor of patients with exfoliation syndrome [80]. A review by Su et al., 2021, reported on the link of LTBP3 dysregulation with various physical developmental disorders in mice and humans [81]. One group demonstrated the compensation of LTBP4 for the loss of LTBP2 in the microfibril formation of mouse embryonic fibroblasts [82]. Furthermore, LTBP4 genetic mutations have been linked to various disorders, including cutis laxa [81,83,84,85], scleroderma [81,86], pulmonary [81,87,88,89], cardiac [81,90,91,92], and cancer [81,93,94]. Our group reported that both TGF-β1 and TGF-β3 modulated LTBP1 expression, but only TGF-β3 modulated LTBP2 expression in 3D HCF constructs. Interestingly, LTBPs were not modulated by exogenous S1P or S1P inhibition (I_2_) [26]. Our current study revealed that fibrotic rescue via stimulation with β1-I_2_ caused a significant downregulation in the expression of LTBPs 1–4; contrastingly, the β1-S1P group caused an upregulation of LTBPs 1–4. Fibrotic prevention via stimulation with I_2_-β1 induced the downregulation of LTBPs 1 and 2, whereas the S1P-β1 group led to the significant upregulation of LTBPs 2 and 4 only. TGF-β activation is dependent on LTBP regulation and release from the large latent complex (LLC), and our findings demonstrated that LTBPs were heavily regulated by S1P prevention and rescue treatments, whereas I_2_ prevention and rescue treatments inhibited their expressions.

TGF-β and S1P overlapping convergence and cell signaling effects have been well documented [62,95], and recent studies involving endometriosis [57], pulmonary fibrosis [58,61], EMT/asthma [59], and renal interstitial fibrosis [60] have reported their involvement in the development of the aforementioned disorders. Although TGF-β and S1P cross-talks have been rigorously investigated in various cells and tissues, their role in the cornea has been understudied and is not yet well understood. Herein, we observed the upregulation of TGF-βRI and II expressions following S1P-β1 and β1-S1P treatment groups but significant downregulation following I_2_-β1 and β1-I_2_ group treatments, indicating that S1P prevention and rescue activated TGF-β receptors, whereas I_2_ prevention and rescue treatments did not.

SMADs are major downstream signaling transducers for TGF-β receptors and have been previously documented for their role in corneal fibrosis [65,66]. Recent studies have reported the impact of fibrosis in response to SMAD inhibition in the cornea. A reduction in corneal fibrosis was observed via the inhibition of SMAD2/3 in human [96,97] and mouse corneas [98]. One study previously demonstrated that murine corneal fibrosis was regulated in part by TGF-β1/SMAD2 activation [99]. Nuwormegbe et al., 2021 [100], revealed that TGF-β1-induced fibrosis was suppressed via SMAD3 signal inhibition in the human cornea. This finding was substantiated by another group, which reported that SMAD3 overexpression enhanced TGF-β1-induced fibroblasts to myofibroblast differentiation in HCFs [101]. Our current study demonstrated pSMAD2/3 and SMAD4 downregulation in response to I_2_-β1 treatment and upregulation following S1P-β1 treatment. Additionally, β1-I_2_ treatment caused the downregulation of pSMAD2 and SMAD4, but β1-S1P led to the upregulation of SMAD4 only.

In the sphingosine rheostat, sphingosine kinase is known as the “fulcrum” due to its critical role in controlling the balance between S1P and ceramide levels [44,46]. Many previous studies have implicated S1P as a fibrotic inducer in various cells and tissues [62]. S1P is generated from ceramide, which is phosphorylated by sphingosine kinases, SphK1 and Sphk2, which can demonstrate oppositional effects. S1P produced by SphK1 in the cytosol can act as a second messenger or can be secreted to bind to S1P receptors and TGF-β receptors, whereas SphK2 resides in the cell nucleus where S1P is generated and regulates gene expression [55]. Recently, SphK1 was found to influence S1P upregulation more than SphK2, and S1PR3 was linked to fibrotic manifestations in the lung [44]. Wang et al., 2023, reported that abnormal S1P content in the circulation affected cardiovascular disorder pathogenesis and S1PR3 mediation of cell proliferation and vascular permeability [45]. Furthermore, the S1PR3 antagonist was observed to improve graft viability in rat heart transplants [102]. Another recent study revealed that S1P-induced epithelial endometriotic cell fibrosis was reliant on S1PR3 activation [103]. Although investigations on S1P in the cornea have been under documented in the past, two recent studies have demonstrated the effects of SphK1/S1P in mouse corneas. Yasuda et al., 2021, revealed that TGF-β1-induced injury increased S1P via SphK1 upregulation modulated by S1PR3 and VEGF-A and angiogenesis [104]. Wilkerson et al., 2022, reported that SphK1 knockout mice had reduced corneal neovascularization following injury [55]. Previously, our group reported that TGF-β1 induced the upregulation of S1PR3 in HCF 3D in vitro constructs [26]. In the current study, we observed the upregulation of S1PR3 following S1P-β1 and β1-S1P treatment groups but a significant downregulation following I_2_-β1 and β1-I_2_ group treatments.

S1P is known to elicit cell and tissue-specific effects but is known largely as an inducer of fibrosis. A recent study observed that S1P stimulation enhanced retinal pigment epithelial cell migration, activated S1PR3, and stimulated αSMA transcription [105]. Meanwhile, another study found that exogenous S1P treatment in human Müller glial cells led to the significant upregulation of α-SMA expression [106]. Yang et al., 2024, found that an S1P agonist reduced cell migration compared to high glucose treatment in rat retinal Müller cells [107]. Our current study found increased cell migration following stimulation with I_2_-β1 and β1-I_2_ treatment groups, with decreased cell migration following stimulation with S1P-β1 and β1-S1P groups after 12 h and 18 h post-scratch. Moreover, we observed reduced fibrosis via αSMA and Collagen III expression regulated by I_2_-β1 and β1-I_2_ treatments.

Our observations demonstrated that S1P complementation to TGF-β1-induced fibrosis led to the activation of SPL and TGF-β pathways, whereas I_2_ treatment inhibited the pathways and resulted in reduced corneal fibrosis. Future investigations would explore the potential involvement of SMAD pathway inhibitors and S1P inhibition as a novel therapy for corneal fibrosis management.

## 4. Materials and Methods

### 4.1. Ethical Approval

Primary human corneal stromal fibroblasts (HCFs) were isolated from human cadaver corneas with no history of ocular or systemic disease and were de-identified prior to analysis. All cadaver corneas were obtained from the National Disease Research Interchange (NDRI, Philadelphia, PA, USA). All studies herein were approved by the North Texas Regional Institutional Review Board (IRB # 2020-030) and adhered to the Declaration of Helsinki.

### 4.2. Human Corneal Fibroblast Cell Isolation and 3D In Vitro Model Cultures

HCFs were isolated from healthy donors by scraping away the epithelium and endothelium, cutting the stromal tissue into 2 × 2 mm pieces, and allowing them to adhere in T25 flasks. The corneal explants were cultured in complete media consisting of Eagle’s Minimum Essential Medium (EMEM: ATCC; Manassas, VA, USA) with 10% fetal bovine serum (FBS: R&D Systems, Minneapolis, MN, USA) and 1% antibiotic-antimycotic (A.A.; Gibco, Life Technologies; Grand Island, NY, USA). HCFs were seeded onto polycarbonate transwell membranes in 6-well plates with 1 × 10^6^ cells/well. The cells were incubated for 24 h hours to allow adherence to the membranes and thereafter were stimulated with 0.5 mM stable vitamin C (0.5 mM 2-O-α-D-glucopyranosyl-L-ascorbic acid [108], Sigma-Aldrich, St. Louis, MO, USA) in a complete medium containing the following treatments: 0.1 ng/mL TGF-β1 (β1), 1 μM sphingosine-1-phosphate (S1P), or 5 μM SPHK I_2_ (I_2_). The TGF-β1 treatment was administered for the first two weeks and was then switched to the S1P or I_2_ treatment for the last two weeks. Additionally, the S1P or I_2_ treatment was administered for the first two weeks and was then switched to the TGF-β1 treatment for the last two weeks. The treatment groups are abbreviated as follows: S1P-β1 (S1P prevention), β1-S1P (S1P rescue), I_2_-β1 (I_2_ prevention), and β1-I_2_ (I_2_ rescue). Constructs with complete media and vitamin C only served as the controls. Fresh treatments were supplied every other day for a total of four weeks. A TGF-β1 stock solution was made at a concentration of 20 μg/mL by dissolving TGF-β1 powder (#240-B; R&D Systems; Minneapolis, MN, USA) in 1 mg/mL bovine serum albumin (BSA) and 4mM HCl. An S1P stock solution was prepared at a concentration of 125 μm by dissolving S1P powder (#860492P; Avanti Polar Lipids; Alabaster, AL, USA) in 4 mg/mL BSA in water at 37 °C inside a glass vessel. A stock solution of SPHK I_2_ (#10009222; Cayman Chemicals; Ann Arbor, MI, USA) was made at a concentration of 5 mM by dissolving the powder in DMSO. Protein was extracted from the 3D constructs for Western blot analysis.

### 4.3. Western Blot Analysis

Protein was extracted from the 3D constructs as previously described [51], and their concentrations and purities were examined using a Pierce™ BCA Protein Assay (ThermoFisher Scientific; Rockford, IL, USA) by measuring absorbance at 562 nm with Gen5 version 3.10 software (BioTek EPOCH2 microplate reader; BioTek; Winooski, VT, USA). The proteins were denatured, added into Novex 4–20% Tris-Glycine Mini Gels (Life Technologies; Carlsbad, CA, USA) at equal concentrations, electrophoresed, and then transferred onto PVDF membranes (Invitrogen, ThermoFisher Scientific; Waltham, MA, USA). The membranes were incubated at room temperature on a shaker for 1 h in a 1X blocking solution (#37565; ThermoFisher Scientific; Rockford, IL, USA). Next, the membranes were incubated overnight at 4 °C in the following primary antibodies: anti-TGF-βRI (ab121024; Abcam; Cambridge, MA, USA), anti-TGF-βRII (ab61213; Abcam; Cambridge, MA, USA), anti-SphK1 (ab302714; Abcam; Cambridge, MA, USA), anti-SphK2 (ab215750; Abcam; Cambridge, MA, USA), anti-S1PR3 (ab126622; Abcam; Cambridge, MA, USA), anti-pSMAD2 (ab53100; Abcam; Cambridge, MA, USA), anti-pSMAD3 (ab52903; Abcam; Cambridge, MA, USA), anti-SMAD4 (ab40759; Abcam; Cambridge, MA, USA), anti-LTBP1 (MBS9603049; MyBioSource; San Diego, CA, USA), anti-LTBP2 (PA551930; Invitrogen; ThermoFisher Scientific; Waltham, MA, USA), anti-LTBP3 (BS72733; Bioworld Technology; St. Louis Park, MN, USA), anti-LTBP4 (MBS9402509; MyBioSource; San Diego, CA, USA), and anti-βactin (ab184092; Abcam; Cambridge, MA, USA). Lastly, the membranes were incubated for 1 h at room temperature in Alexa Flour^®^ 488 Goat anti-Rabbit secondary antibody (A-11008; Life Technologies; Carlsbad, CA, USA). Primary and secondary antibody dilutions were used as recommended by the manufacturers. The iBright 1500 FL imaging system (ThermoFisher Scientific; Rockford, IL, USA) was used for fluorescent signal detection. The membranes were stripped and reprobed with primary antibodies as needed. Pre-conjugated anti-βactin housekeeping antibody was used to adjust all target values, and their fold expressions were plotted. All experimental conditions were repeated 3 times.

### 4.4. 2D Scratch Assay—Cell Migration

HCFs were seeded at a density of 5 *×* 10^5^ cells/well in 12-well plates in a control medium. Following 24 incubations, a scratch was administered through the confluent cell layer using a 10 μL pipette tip, and cell migration was observed at 0, 12, 18, and 24 h post-scratch. The TGF-β1 treatment was administered for the first 6 h and the S1P or I_2_ treatment was administered for the last 6 h. Additionally, the S1P or I_2_ treatment was administered for the first 6 h and the TGF-β1 treatment was administered for the last 6 h. An EXI-310 inverted microscope (Accu-Scope Inc.; Commack, NY, USA) was used to capture images of the wound closure progression. Cell migration was measured and quantified using ImageJ 1.53e software. All experimental conditions were repeated 5 times.

### 4.5. Statistical Analysis

All data were reported as mean ± SEM. GraphPad Prism 9.4.1 (GraphPad Prism; San Diego, CA, USA) software was used to calculate statistically significant differences using one-way ANOVA, where *p* < 0.05 was considered statistically significant: * *p* < 0.05, ** *p* < 0.01, *** *p* < 0.001, and **** *p* < 0.0001.

## Data Availability

The data in this manuscript study are available upon request from the corresponding author.
